# MicroRNA expression profiling with a droplet digital PCR assay enables molecular diagnosis and prognosis of cancers of unknown primary

**DOI:** 10.1002/1878-0261.13026

**Published:** 2021-06-23

**Authors:** Noemi Laprovitera, Mattia Riefolo, Elisa Porcellini, Giorgio Durante, Ingrid Garajova, Francesco Vasuri, Ariane Aigelsreiter, Nadia Dandachi, Giuseppe Benvenuto, Federico Agostinis, Silvia Sabbioni, Ioana Berindan Neagoe, Chiara Romualdi, Andrea Ardizzoni, Davide Trerè, Martin Pichler, Antonietta D'Errico, Manuela Ferracin

**Affiliations:** ^1^ Department of Experimental, Diagnostic and Specialty Medicine (DIMES) University of Bologna Italy; ^2^ Department of Life Sciences and Biotechnologies University of Ferrara Italy; ^3^ Pathology Unit IRCCS Azienda Ospedaliero‐Universitaria di Bologna Italy; ^4^ Medical Oncology Unit University Hospital of Parma Italy; ^5^ Diagnostic and Research Institute of Pathology Medical University of Graz Austria; ^6^ Division of Oncology Medical University of Graz Austria; ^7^ Department of Biology University of Padua Italy; ^8^ Research Center for Functional Genomics, Biomedicine and Translational Medicine “Iuliu Hatieganu” University of Medicine and Pharmacy Cluj‐Napoca Romania; ^9^ Division of Medical Oncology IRCCS Azienda Ospedaliero‐Universitaria di Bologna Italy

**Keywords:** cancer of unknown primary, droplet digital PCR, metastasis, microRNAs, molecular diagnostics

## Abstract

Metastasis is responsible for the majority of cancer‐related deaths. Particularly, challenging is the management of metastatic cancer of unknown primary site (CUP), whose tissue of origin (TOO) remains undetermined even after extensive investigations and whose therapy is rather unspecific and poorly effective. Molecular approaches to identify the most probable TOO of CUPs can overcome some of these issues. In this study, we applied a predetermined set of 89 microRNAs (miRNAs) to infer the TOO of 53 metastatic cancers of unknown or uncertain origin. The miRNA expression was assessed with droplet digital PCR in 159 samples, including primary tumors from 17 tumor classes (reference set) and metastases of known and unknown origin (test set). We combined two different statistical models for class prediction to obtain the most probable TOOs: the nearest shrunken centroids approach of Prediction Analysis of Microarrays (PAMR) and the least absolute shrinkage and selection operator (LASSO) models. The molecular test was successful for all formalin‐fixed paraffin‐embedded samples and provided a TOO identification within 1 week from the biopsy procedure. The most frequently predicted origins were gastrointestinal, pancreas, breast, lung, and bile duct. The assay was applied also to multiple metastases from the same CUP, collected from different metastatic sites: The predictions showed a strong agreement, intrinsically validating our assay. The final CUPs' TOO prediction was compared with the clinicopathological hypothesis of primary site. Moreover, a panel of 13 miRNAs proved to have prognostic value and be associated with overall survival in CUP patients. Our study demonstrated that miRNA expression profiling in CUP samples could be employed as diagnostic and prognostic test. Our molecular analysis can be performed on request, concomitantly with standard diagnostic workup and in association with genetic profiling, to offer valuable indications about the possible primary site, thereby supporting treatment decisions.

## Graphical abstract

Cancer of unknown primary site (CUP) patients suffer the burden of a metastatic disease whose site of origin is unidentifiable, even after intensive clinical investigations. The lack of a recognized primary site limits patients' treatment options. In this manuscript, we present a molecular assay, based on digital miRNA profiling, that allowed the prediction of the primary site of 53 CUPs.

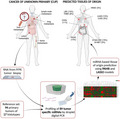

**Abbreviations**
BLCA: transitional cell carcinoma of bladder
BRCA: breast invasive carcinoma
CHOL: cholangiocarcinoma
CRC: colorectal adenocarcinoma
CUP: cancer of unknown primary site
ddPCR: droplet digital PCR
FFPE: formalin‐fixed, paraffin‐embedded
GEP: gene expression profiles
GI‐NET: gastrointestinal neuroendocrine carcinoma
HE: hematoxylin–eosin
HNSC: head and neck squamous cell carcinoma
HPV: human papillomavirus
HR: hazard ratio
IHC: immunohistochemistry
KICA: kidney renal clear and renal papillary cell carcinoma
KIRC: kidney renal clear cell carcinoma
KIRP: kidney renal papillary cell carcinoma
LASSO: least absolute shrinkage and selection operator
LBC: luminal nonspecial type and lobular breast carcinoma
LIHC: hepatocellular carcinoma
LUAD: lung adenocarcinoma
LUSC: lung squamous cell carcinoma
ND: not defined
NSC: nearest shrunken centroids
OS: overall survival
OV: ovarian serous carcinoma
PAAD: pancreas exocrine adenocarcinoma
PAMR: Prediction Analysis of Microarrays for R
PPR: positive prediction rate
PRAD: prostate adenocarcinoma
ROC: receiver operating characteristic
SKCM: melanoma of skin
STAD: gastric adenocarcinoma
TCGA: The Cancer Genome Atlas
TGSC: germ cell seminomatous carcinoma
TNBC: triple-negative breast cancer
TOO: tissue of origin
UCEC: endometrial adenocarcinoma

## 1 Introduction

Cancer of unknown primary origin (CUP) describes newly diagnosed tumors presenting as metastatic cancers, whose primary site cannot be identified after detailed standardized physical examinations, blood analyses, imaging, and immunohistochemical (IHC) testing [1]. CUP biology represents a real riddle, and several theories have been proposed to describe CUP origin. According to the two prevailing hypotheses, CUPs could originate from small undetectable, dormant, or later regressed primary lesions or represent early disseminating, aggressive metastatic entities with no existing primary site [1, 2]. A comprehensive genetic and transcriptomic analysis of multiple metastases from the same CUP patient revealed an unusually high level of similarity, suggesting a simultaneous origin [3].

Postmortem investigations on CUP patients reported the identification of a primary tumor in about 75% of cases and highlighted the prevalent epithelial origin of CUPs. The most common primary sites were represented by lung, pancreas, hepatobiliary tract, kidney, colon, genital organs, and stomach [4]. Population‐based studies reported decreasing trends of CUP incidence in different countries in the last decade, possibly as a consequence of novel diagnostic techniques that improved primary site identification or a more consequent and widespread approach to follow standardized diagnostic workup guidelines [5]. Nonetheless, incidence rates still vary among different countries worldwide.

International guidelines for tumor treatment are essentially based on primary site indication. Therefore, CUP treatment requires a rather unspecific blind approach, which is very challenging for the treating physicians. As a consequence, CUPs are usually treated with empiric platinum‐based chemotherapy regimens that are poorly effective. CUP patients have a short life expectancy (average overall survival 4–9 months, 20% survive more than 1 year) that have not improved in the last decades. In the most recent CUP NCCN guidelines (v.2/2020), there are 11 different chemotherapy regimens indicated for adenocarcinoma and nine for squamous histology. However, these regimens remain empirical since they are mostly based on single‐arm phase II clinical trials [6, 7, 8] and small randomized prospective trials [9, 10, 11]. In addition, the lack of primary tumor definition prevents most patients to be treated in clinical practice with novel, very effective treatment such as immunotherapy or molecular targeted therapies for which current registered indications are mostly disease‐oriented. Finally, patients with occult primary tumors suffer a great psychological burden of an unidentified disease. The use of molecular tests that could identify the most probable site of origin or an approach based on personalized medicine may be useful to assist in the selection of the best treatment options and potentially improve CUP prognosis and survival.

The identification of druggable alterations in CUP tumors could improve the otherwise limited treatment options. Recently, several studies focused on the analysis of CUP mutational profiles [12, 13, 14]. A comprehensive retrospective analysis, using the 236‐gene FoundationOne assay (Roche Foundation Medicine, Cambridge, MA, USA), explored the genomic profiles of 200 CUPs [13]. At least one clinically relevant genetic alteration was found in 96% of CUPs, with a mean of 4.2 alterations per tumor. The most frequently mutated genes were *TP53* (55%), *KRAS* (20%), *CDKN2A* (19%), *MYC* (12%), *ARID1A* (11%), and *MCL1* (10%). According to this study, potentially druggable mutations were discovered in 20% of CUPs. Varghese *et al*. [14] identified the actionable mutations in a dataset of 150 CUPs analyzed with the MSK‐IMPACT panel and in another dataset of 200 CUPs from Ross *et al*. [13]. Potentially druggable alterations were present in 30% of CUP cases (FDA level 2–3 of evidence for actionability) [14].

Another way to improve the choice of CUP therapeutic options is the prediction of CUP site of origin using molecular assays. This strategy is based on the observation that metastatic tumor cells retain some molecular characteristics of the tissue of origin, despite going through de‐differentiation and epithelial–mesenchymal transition programs. This tissue‐specific molecular signature can be leveraged to infer CUPs' sites of origin. In the past decade, several molecular classifiers were developed. These classifiers were built based on gene expression profiles (GEP) [15, 16, 17, 18], microRNAs [19, 20], or DNA methylation [21, 22, 23].

A number of studies reported evidences in favor of this hypothesis, showing a prolonged survival in patients treated with cancer‐specific agents compared to standard chemotherapy [22, 24, 25, 26, 27]. Results from a prospective study on nearly 300 patients with CUP who were treated according to GEP molecular prediction revealed a significant increase in median survival time (12.5 months) [28].

In addition, GEP proved a higher diagnostic accuracy compared to standard immunohistochemistry (IHC) staining in the identification of CUP primary site, especially in moderately or poorly differentiated cases [28, 29]. The most recent NCCN CUP guidelines [30] support the use of gene expression profiling to get a diagnostic benefit in CUP management, though the achievement of a clinical benefit still needs to be determined. Results from the phase III clinical trial NCT03278600 could help to clarify the value of tissue‐of‐origin profiling in predicting primary site and directing therapy in CUP patients.

However, the analysis of GEP in archival formalin‐fixed, paraffin‐embedded (FFPE) tissues is limited by the quality of extracted RNA, which is usually low. Thus, the reported rate of technical success of GEP assays (i.e., CancerTypeID assay) is 85% [25]. On the contrary, microRNAs (miRNAs) are robustly detected irrespective of the quality of the tissue sample [31, 32] and are highly stable and resistant to RNAase degradation either in compromised archived clinical specimens [33, 34] or in biological fluids [35]. Molecular miRNA profiling of FFPE samples could be successfully obtained from all the available samples [19, 36].

Independently from the molecular assay choice, assessing the true clinical benefit of molecular profiling is challenging because it relies on surrogate measures (correlation with IHC findings, clinical presentation or response to therapy), given that a real primary site identification is seldom available.

In a previous microarray‐based study, we identified a cancer type‐specific miRNA signature able to predict metastatic tumor tissue of origin of CUPs among 10 possible primary sites [19]. This predictive tool was employed in a few occasions to provide clinicians with indications of a possible primary site [37]. However, microarray technology limitations prevent the execution of such analysis on a routine basis. To extend the analysis to more tumor types and overcome the technical limits of microarray technology, we developed a miRNA‐based molecular assay for a rapid, on‐demand molecular tumor characterization and primary site prediction [38]. Unlike previous assays, our test employs droplet digital PCR (ddPCR) technology to assess the absolute level of a predetermined set of 89 miRNAs in FFPE tumor tissues. This assay is applied here to predict the most probable primary tissue(s) of a set of 53 cancers of unknown or uncertain origin, obtaining a broad spectrum of primary site predictions with different levels of confidence.

## 2 Methods

### 2.1 Patients and tumor samples

A total number of 159 FFPE samples from 150 patients were collected for this study. Patients were diagnosed and treated at Sant'Orsola‐Malpighi Bologna University Hospital, Italy (*N* = 84), at the University Hospital of Ferrara, Italy (*N* = 52), or at the Medical University of Graz, Austria (*N* = 14). The study cohort consists of patients with tumors with a clearly recognized primary site (*N* = 104 patients, *N* = 106 samples) and patients with cancer of unknown or uncertain origin (CUPs, *N* = 46 patients, *N* = 53 samples). A summary of samples and patients enrolled in the study is reported in Table 1. Primary tumors included samples obtained from the following tumor sites/types: lung (LUAD, adenocarcinoma, *N* = 6 and LUSC, squamous cell carcinoma, *N* = 3), pancreas (PAAD, exocrine adenocarcinoma, *N* = 5), ovary (OV, ovarian serous carcinoma, *N* = 6), liver (LIHC, hepatocellular carcinoma, *N* = 6), biliary tract (CHOL, cholangiocarcinoma, *N* = 6), kidney (KICA, which includes kidney renal clear cell carcinoma or KIRC, *N* = 5 and kidney renal papillary cell carcinoma or KIRP, *N* = 3), colorectum (CRC, adenocarcinoma, *N* = 7), testis (TGSC, germ cell seminomatous carcinoma, *N* = 4), endometrium (UCEC, adenocarcinoma, *N* = 5), stomach (STAD, adenocarcinoma, *N* = 5), bladder (BLCA, transitional cell carcinoma, *N* = 4), breast (LBC, luminal nonspecial type and lobular breast carcinoma, *N* = 5), triple‐negative breast cancer (TNBC, *N* = 3), prostate (PRAD, adenocarcinoma, *N* = 5), melanoma (SKCM, melanoma of skin, *N* = 7), head and neck (HNSC, squamous cell carcinoma, *N* = 6), and gastrointestinal neuroendocrine carcinoma (GI‐NET, *N* = 5). We assessed 10 metastases of known origin, derived from lung, melanoma, stomach, prostate, head and neck, kidney, colon, breast, pancreas, and endometrium. A total number of 53 CUP samples were included in this study, specifically 43 retrospective and 10 prospective cases. Moreover, from five retrospective CUP patients we were able to obtain metastatic biopsies collected from multiple sites that were independently analyzed. CUP diagnosis was obtained after detailed clinical and pathological investigations. For each sample, a full IHC panel was assessed at the time of diagnosis and the outcome was recorded. However, we need to underline that our collection of CUP samples is heterogeneous since it derives from patients that received the diagnosis in different time; specifically, 14 of them (26%) received the diagnosis of CUP between 2005 and 2009, 26 between 2010 and 2014 (49%), and 13 between 2015 and 2019 (25%).

**Table 1:** Summary of samples and patients enrolled in the study. BLCA, transitional cell carcinoma of bladder; CHOL, cholangiocarcinoma; CRC, colorectal adenocarcinoma; GI‐NET, gastrointestinal neuroendocrine carcinoma; HNSC, head and neck squamous cell carcinoma; KIRC, kidney renal clear cell carcinoma; KIRP, kidney renal papillary cell carcinoma; LBC, luminal nonspecial type and lobular breast carcinoma; LIHC, hepatocellular carcinoma; LUAD, lung adenocarcinoma; LUSC, lung squamous cell carcinoma; OV, ovarian serous carcinoma; PAAD, pancreas exocrine adenocarcinoma; PRAD, prostate adenocarcinoma; SKCM, melanoma of skin; STAD, gastric adenocarcinoma; TGSC, germ cell seminomatous carcinoma; TNBC, triple‐negative breast cancer; UCEC, endometrial adenocarcinoma; ND, not defined.

Characteristics		Primaries		Metastases		CUPs	
		*n*	%	*n*	%	*n*	%
Patients	*n* = 150	94		10		46	
Prospective						10	22
Retrospective		94		10		36	78
Samples	*n* = 159	96		10		53	
Sex							
Male	78	48	50	3	30	26	49
Female	64	32	33	5	50	27	51
ND	17	16	17	2	20	0	0
Age, years							
Median		66		71		67	
Range		44–85		60–86		42–87	
ND		62		4		0	
Primary tumor classes							
BLCA		4					
CHOL		6					
CRC		7		1			
GI‐NET		5					
HNSC		6		1			
KIRC		5		1			
KIRP		3					
LBC		5		1			
LIHC		6					
LUAD		6		1			
LUSC		3					
OV		6					
PAAD		5		1			
PRAD		5		1			
SKCM		7		1			
STAD		5		1			
TGSC		4					
TNBC		3					
UCEC		5		1			
Metastatic sites							
Bone						2	
Bone marrow						1	
Brain				1		2	
Breast						3	
Cerebellum						1	
Colon				1		1	
Dermis						1	
Duodeno						1	
Kidney						1	
Liver				4		12	
Lung				1		2	
Lymph node						14	
Muscle						1	
ND						1	
Pericardium				1			
Pleura						5	
Prostate						2	
Skin				1			
Soft tissues						2	
Stomach				1			
Thyroid						1	

For each sample, 10 µm thick tissue sections (*N* = 2–5) were obtained. The first section was stained with hematoxylin–eosin (HE) and examined by an expert pathologist to select the tumor area, which was grossly dissected before RNA extraction. Tumor cell fraction was evaluated to select samples with at least 30% cellularity. The study was conducted in accordance with the Declaration of Helsinki, and the protocol was approved by the Ethics Committee Center Emilia‐Romagna Region—Italy (protocol 130/2016/U/Tess), and Medical University of Graz (vote no. 30‐520 ex 17/18). Prospective patients provided written informed consent. Detailed pathological characteristics of cancer patients are available in Tables 2 and S1.

**Table 2:** Prediction outcome in cancer of unknown primary site. BLCA, transitional cell carcinoma of bladder; CHOL, cholangiocarcinoma; GI‐NET, gastrointestinal neuroendocrine carcinoma; HNSC, head and neck squamous cell carcinoma; KICA, kidney renal clear cell and papillary cell carcinoma; LBC, luminal non‐special type and lobular breast carcinoma; LIHC, hepatocellular carcinoma; LUAD, lung adenocarcinoma; LUSC, lung squamous cell carcinoma; ND, not defined; OV, ovarian serous carcinoma; PAAD, pancreas exocrine adenocarcinoma; STAD‐CRC, colorectal and gastric adenocarcinoma; TNBC, triple negative breast cancer.

Sample ID	Sex	Multiple metastases	Age at diagnosis	Tumor cellularity (%)	Status	Biopsy site	Histotype	K WS	K7	K20	Other IHC testing	Pathological hypothesis	Clinical hypothesis	Late identification of the primary site	Molecular prediction^a^
CB002	F		65	50	Retrospective	Liver	Adenocarcinoma	ND	POS	NEG	CA125^+^, chromogranin^−^, ER^−^, GATA3^−^	ND	ND		LBC
CB003	F		81	60	Retrospective	Lymph node	Carcinoma	POS	ND	ND	S100^−^, CD10^−^, TTF1^−^, ER^−^, PR^−^, HMB45^−^, GATA3^−^	ND	ND		HNSC
CB011	F		75	85	Retrospective	Lymph node	Carcinoma	POS	POS	ND	WT1^+^, vimentin ^+^, chromogranin^+/−^, synaptophysin^+/−^, calretinin^−^, CD10^−^, ER^−^, PR^−^, p63^−^, CD45^−^, CDX2^−^	Mullerian or kidney	ND		GI‐NET
CB012	M		76	30	Retrospective	Bone marrow	Adenocarcinoma	POS/NEG	NEG	NEG	S100^+^, PSA^+/−^, CK14^−^, CK18^−^, CK 19^−^, ER^−^, PR^−^, HMB45^−^, MUC1^+^, TTF1^−^, vimentin^−^	Prostate	Melanoma		CHOL^b^
CB013	F		71	70	Retrospective	Lymph node	Mucinous adenocarcinoma	POS	NEG/POS	NEG	ER^−^, PR^−^, TTF1^−^, CDX2^−^	Gastrointestinal	ND		STAD‐CRC
CB014	F		47	50	Retrospective	Lymph node	Papillary adenocarcinoma	POS/NEG	POS/NEG	NEG	vimentin^+/−^, actin^−^, CK14^+^, ER^−^, PR^−^, HER2^−^, TTF1^−^, thyroglobulin^−^, WT1^−^, GATA3^+^, P40^−^, PAX8^−^	TN breast or thyroid	ND		TNBC
CB033	F		77	60	Prospective	Lymph node	Carcinoma	POS	POS	NEG	CK 5^−^6^+^, CK14^+^, GATA3^+/−^, ER^−^, PR^−^, AR, HER2^−^, P63^+^, P40^+^, synaptophysin^−^, PAX8^−^, napsin A^−^ WT1^−^, TTF1^−^	Sudoriparous gland	Breast		LBC
CB053	F		60	75	Prospective	Liver	Adenocarcinoma	ND	POS	NEG	TTF1^+^, ALK^−^, KRAS G34T (pyrosequencing)	Lung	ND		STAD‐CRC
CB054	F		59	70	Prospective	Lung	Carcinoma	POS	ND	ND	GATA3^+^, ER^−^, PR^−^, HER2^−^, Ki67 96%, p40‐, p63^−^, TTF1^−^, CDX2^−^	Breast	ND	LBC	LBC
CB055	F		49	50	Prospective	Lymph node	Adenocarcinoma	POS	POS	POS	CDX2^+^, chromogranin^−^, synaptophysin^−^, CD56^−^, HER^−^2^−^, MSI^−^	Gastrointestinal	ND		CHOL
CB061	M		60	70	Retrospective	Kidney	Adenocarcinoma	POS	POS	ND	AR^+^, CD10^−^, OCT4^−^, PSA^−^, RCC^−^, TTF1^−^, GATA3^−^, NKX34.1^−^	Breast or kidney	ND		LBC
CB062	M		87	80	Prospective	Seminal vesicle	Carcinoma	POS	NEG	NEG	CDX2^+^, SMA^−^, CD34^−^, desmin^−^, HER2^−^, Ki67:50%, MART1^−^, MSI^−^, NKX3.1^−^, S100^−^	Gastrointestinal	ND		CHOL/LBC/KICA
CB064	M		58	65	Prospective	Liver	Squamous carcinoma	ND	NEG	NEG	P63^+^, CK14^+^, chromogranin^−^, synaptophysin^−^, CD45^−^, S100^−^	ND	ND		TNBC
CB071	F		64	60	Prospective	Liver	Carcinoma	ND	POS	NEG	CEA^+/−^, GATA3^−^, ER^−^, PR^−^/^+^, TTF1^−^, PAX8^−^	Biliary duct	ND	CHOL	CHOL
CB090	F		63	30	Prospective	Duodeno	Adenocarcinoma	ND	POS	NEG	CDX2^+/−^, MUC 1^+^, MUC 2^−^, MUC 5AC^+^, MUC 6^+/−^, HER2^−^, MSI^−^, synaptophysin^−^, TTF1^−^	Gastrointestinal	ND		STAD‐CRC^b^
CB095	F		70	80	Prospective	Soft tissue	Squamous carcinoma	ND	POS	NEG	CA125^−^, CA15.3^−^, CDX2^+/−^, desmin^−^, ER^−^, GATA3^−^, MUC1^+^, P40^−^, PAX^−^8^−^, PD^−^L1 70%, ER^−^, S100^−^, synaptophysin^−^, TTF1^−^	ND	ND		BLCA
CB097	F		68	90	Retrospective	Liver	Neuroendocrine	ND	ND	ND	chromogranin ^+^, synaptophysin ^+^, CDX2^−^, gastrin^−^, glucagon^−^, insulin^−^, TTF1^−^	Gastrointestinal NET	Liver		PAAD
CB098	F	B01	42	80	Retrospective	Lymph node	Squamous cell carcinoma	POS	POS	POS	CD10^−^, CEA^+^, GATA3^−^, HER2^−^, Ki67 90%, Mammaglobin^−^, ER^−^, PR^−^, AR^−^, HER2^−^, BRAF V600E^−^	Breast	Breast	TNBC	TNBC
CB100	F	B02	42	40	Retrospective	Lymph node	Squamous cell carcinoma	POS	POS	POS	CD10^−^, CEA^+^, GATA3^−^, HER2^−^, Ki67 90%, Mammaglobin^−^, TTF1^−^, ER^−^, PR^−^, AR^−^, HER2^−^, BRAF V600E^−^, EBV^−^ (ISH)	Breast	Breast	TNBC	LUAD/TNBC^b^
CB101	F	B03	42	80	Retrospective	Breast	Adenocarcinoma	POS	POS	POS	CD10^−^, CEA^+^, GATA3^−^, HER2^−^, Ki67 70%, Mammaglobin^−^, Cathepsin K^−^, ER^−^, PR^−^, AR^−^, HER2^−^, BRAF V600E^−^, EBV^−^ (ISH)	Breast	Breast	TNBC	TNBC
CB102	F	B04	42	85	Retrospective	Breast	Adenocarcinoma	POS	POS	POS	CD10^−^, CEA^+^, GATA3^−^, HER2^−^, Ki67 60%, Mammaglobin^−^, Cathepsin K^−^, ER^−^, PR^−^, AR^−^, HER2^−^, BRAF V600E^−^, EBV^−^ (ISH)	Breast	Breast	TNBC	LBC
CB103	M		81	50	Retrospective	Lymph node	Large cell carcinoma	POS	POS	POS	CK14^+^, MUC1^+^, TTF1^−^, CD10^−^, CD117^−^, CD56^−^, Ki67 50%, EBV^−^ (ISH)	Lung	ND		LUAD
CB104	M		43	90	Retrospective	Prostate	Squamous cell carcinoma	POS	ND	NEG	PSA^−^, P40^+^	Prostate or bladder	Bladder		LUSC
CB105	M	E01	61	40	Retrospective	Liver	Adenocarcinoma	ND	POS	NEG	CDX2^−^, MUC1^+^, MUC2^+^, TTF1^−^, HER2^−^	Gastrointestinal	Gastrointestinal		STAD‐CRC^b^
CB106	M	E02	61	70	Retrospective	Thyroid	Neuroendocrine	ND	POS	NEG	CD56^−^, CDX2^−^, chromogranin^+^, MUC1^+^, MUC2^+^, synaptophysin^−^, TTF1^−^	Gastrointestinal	Gastrointestinal		GI‐NET, STAD‐CRC
CB108	M	F01	74	80	Retrospective	Bone	Adenocarcinoma	POS	POS	NEG	PSA^−^, TTF1^−^, CD34^−^	Upper gastrointestinal tract	Upper gastrointestinal tract		LBC/ STAD‐CRC^b^
CB109	M	F02	74	65	Retrospective	Dermis	Undifferentiated carcinoma	POS	POS	NEG	CD31^−^, CD34^−^, CK 5/6^−^, Factor VIII^−^, LCA^−^, PSA^−^, S100^−^, SOX9^−^, TTF1^−^, vimentin^−^	Upper gastrointestinal tract	ND		STAD‐CRC
CB110	F		57	70	Retrospective	Brain	Adenocarcinoma	POS	POS	NEG	BRAF V600E^−^, TTF1^−^	Mullerian	Lung		OV
CB112	M		79	35	Retrospective	Colon	Adenocarcinoma	POS	POS	NEG	CDX2^−^, calretinin^−^, CDX2^−^, CEA^−^, PDPN^−^, TTF1^−^	ND	ND		LUAD^b^
CB115	F		86	50	Retrospective	Muscle	Adenocarcinoma	POS	POS	NEG	TTF1^−^, CDX2^−^	Intrahepatic bile duct	ND		STAD^−^CRC/PAAD
CB116	F		65	60	Retrospective	ND	Adenocarcinoma	ND	POS	NEG	TTF1^−^, CDX2^−^	Extrahepatic bile duct	ND		STAD‐CRC
CB117	M		69	80	Retrospective	Lymph node	Carcinoma	POS	ND	ND	HMB45^−^, MART1^−^, S100^−^, CD10^−^	ND	ND		LUAD
CB118	M		61	60	Retrospective	Lymph node	Adenocarcinoma	ND	POS	NEG	CDX2^−^, TTF1^−^	Pancreas	Lung		LUAD
CB119	M	Q01	66	50	Retrospective	Lymph node	Adenocarcinoma	POS	ND	ND	CK 5/6^−^, Ki67 50%, MUC1^+^, PSA^−^	Prostate or bladder	ND		HNSC
CB120	M	Q02	66	50	Retrospective	Lymph node	Adenocarcinoma	POS	ND	ND	CK 5/6^−^, Ki67 50%, MUC1^+^, PSA^−^	Prostate or bladder	ND		HNSC/LUAD
CB121	M	R01	69	30	Retrospective	Bone	Adenocarcinoma	POS	NEG	POS	BRAF V600E^−^, CDX2^+^, PSA^−^, TTF1^−^	Gastrointestinal	Bile duct		CHOL^b^
CB122	M	R02	69	65	Retrospective	Liver	Adenocarcinoma	POS	NEG	POS	BRAF V600E^−^, CDX2^+^, PSA^−^, TTF1^−^	Gastrointestinal	Bile duct		CHOL/PAAD
CB125	M		64	60	Prospective	Cerebellum	Mucinous adenocarcinoma	ND	NEG	ND	PDL1^−^, TTF1^−^	ND	Gastrointestinal or lung		STAD‐CRC
PF005	F		75	ND	Retrospective	Lung	Poorly differentiated adenocarcinoma	ND	ND	ND	ND	ND	ND		LBC
PF006	F		81	ND	Retrospective	Brain	Clear cell carcinoma	ND	ND	ND	ND	Kidney	Lung		LUAD
PF007	M		53	ND	Retrospective	Liver	Adenocarcinoma	ND	ND	ND	ND	Gastrointestinal	ND		STAD‐CRC
PF011	M		75	ND	Retrospective	Lung	Carcinoma with a transitional/squamous and glandular differentiation	ND	ND	ND	TTF1^−^	ND	ND		LUSC
PF013	F		71	ND	Retrospective	Liver	Poorly differentiated adenocarcinoma	ND	ND	ND	ND	ND	Esophagus		CHOL
PF017	M		72	ND	Retrospective	Liver	Adenocarcinoma	ND	ND	ND	ND	ND	Gallbladder		PAAD
PF018	F		79	ND	Retrospective	Liver	Adenocarcinoma	ND	ND	ND	ND	Pancreas	Small intestine		STAD‐CRC
PF019	M		74	ND	Retrospective	Liver	Adenocarcinoma	ND	ND	ND	ND	Pancreas	Pancreas		PAAD/LBC
PF020	M		72	ND	Retrospective	Pleura	Adenocarcinoma	ND	ND	ND	ND	ND	Lung		PAAD
PF021	F		79	ND	Retrospective	Pleura	Adenocarcinoma	ND	POS	ND	TTF1^+^, CEA^+^, ER^−^, PR^−^	Lung	NA		PAAD
PF022	M		73	ND	Retrospective	Pleura	Adenocarcinoma	ND	ND	ND	CEA^+^, TTF1^−^	ND	Gastrointestinal		PAAD
PF024	M		77	ND	Retrospective	Pleura	Adenocarcinoma	ND	ND	ND	TTF1^+^	Lung	NA		LUSC
PF025	M		50	ND	Retrospective	Pleura	Adenocarcinoma	ND	ND	ND	ND	ND	Lung		LIHC
PF059	F		73	ND	Retrospective	Lymph node	Poorly differentiated carcinoma	POS	POS/NEG	NEG	TTF1 ^−^, P63^−^, CD45 ^−^, CDX2 ^−^, MART1^−^, S100 ^−^, ER^+/−^, PR ^−^, HER2^−^ , Ki67 85%, EBV^−^ (ISH)	Breast	ND		LUAD
PF080	F		52	ND	Retrospective	Lymph node	Adenocarcinoma	ND	POS	POS	CDX2^+^, ER^+^, PR^+^, CA125^−^, CD10^−^, CEA^+^, chromogranin^−^, NSE^−^, TTF1^−^, vimentin^−^, WT1^−^	Breast or genital system	ND		PAAD

^a^ The most probable primary sites according to the above‐mentioned criteria

^b^ Suboptimal sample deriving from bone metastases or with a tumor cellularity ≤ 40%.

### 2.2 Ethics approval and consent to participate

The study was conducted in accordance with the Declaration of Helsinki, and the protocol was approved by the Ethics Committee Center Emilia‐Romagna Region—Italy (protocol 130/2016/U/Tess), and Medical University of Graz (vote no. 30‐520 ex 17/18). Prospective patients provided written informed consent.

### 2.3 RNA extraction and cDNA conversion

Total RNA, including microRNAs, was isolated from the tumor FFPE sections using miRNeasy FFPE kit (Qiagen, Hilden, Germany, Cat No. 217504; miRNeasy FFPE Handbook Qiagen, HB‐0374‐005). We followed the protocol: *Purification of Total RNA, Including miRNA, from FFPE Tissue Sections* in Qiagen miRNeasy FFPE Handbook (v. January 2020) and the Appendix A protocol: *Deparaffinization using xylene, limonene or CitriSolv* for deparaffinization. RNA was eluted in 20–30 µL of nuclease‐free water and frozen at −80 °C. RNA yield and quality were assessed with NanoGenius Spectrophotometer (ONDA Spectrophotometer, Giorgio Bormac s.r.l., Carpi, Italy). All samples were suitable for the molecular testing.

RNA conversion to cDNA was performed using the miRCURY LNA RT Kit (Qiagen, Cat No. 339340; miRCURY LNA miRNA PCR Handbook, HB‐2431‐002). The 10 µL reaction mix was prepared for each sample mixing: 2 μL of 5× reaction buffer, 4.5 μL of nuclease‐free water, 1 μL of enzyme mix, 0.5 μL of UniSp6 RNA spike‐in, and 2 μL of diluted RNA (10 ng of total RNA). The resulting cDNA was stored in LoBind DNA Eppendorf tubes (Eppendorf, Hamburg, Germany, 0030108051) at −20 °C. For each sample, a RT‐qPCR was performed as quality control step using miRCURY LNA miRNA PCR Assays (Qiagen) to test UniSp6 (Cat No. YP00203954) and SNORD44 (Cat No. YP00203902) targets. UniSp6 threshold cycle (Ct) informs about the RT reaction efficiency. SNORD44 was tested to assess RNA integrity and amplifiability and to establish the cDNA dilution prior to digital droplet PCR (ddPCR) analysis. For SNORD44 Ct ranging 24–30 (threshold set at 160), cDNA was diluted 1 : 50; for Ct below 24, cDNA was diluted 1 : 100–1 : 200; and when Ct was higher than 30, the RT was repeated again using undiluted RNA and qPCR analysis repeated. cDNA was further diluted 1 : 10 in miR‐21‐5p and UniSP6 wells. Applying these criteria, we prevented ddPCR saturation problems or low miRNA expression levels in ddPCR analysis.

### 2.4 MicroRNA selection

We implemented a miRNA signature for tumor primary site prediction integrating two published signatures [19, 39] plus 10 additional miRNAs (miR‐661, miR‐649, miR‐24‐3p, miR‐16‐5p, miR‐320a, miR‐224‐5p, miR‐423‐5p, miR‐25‐3p, miR‐331‐3p, and miR‐103a‐3p) as detailed in Table S2. Specifically, the first microarray‐based molecular study [39] analyzed up to 25 different histological subtypes to identify a 48‐miRNA signature that was able to efficiently infer the site of origin when applied on metastases of known origin; similarly, the second study [40] comprehended 10 tumor classes in the training set and identified a 47‐miRNA signature that proved its ability to discriminate the tissue of origin in metastases of known origin and was also applied on CUPs. The additional miRNAs we decided to include in the panel were selected as candidate reference miRNAs or to widen the number of the assessed miRNAs, with the aim to test this tool on novel tumor classes or histotypes (CHOL, TGSC, TNBC, and GI‐NET), not included in the two previously mentioned studies.

### 2.5 Droplet digital PCR and data analysis

Prespotted custom plates (96‐well format) were designed to comprehend 89 different miRCURY LNA miRNA primers (Qiagen), three assays for small nuclear or nucleolar RNAs as reference candidates (SNORD44, SNORD48, and snRNAU6), two interplate calibrator assays (UniSp3), a control plate assay (UniSP6), and a no template control (NTC) as described in [38] (miRNA list and plate set up in Table S2).

EvaGreen‐based droplet digital PCR was performed as described in Refs [38, 41, 42]. Thermal cycling conditions were as follows: 95 °C for 5 min, then 40 cycles of 95 °C for 30 s and 58 °C for 1 min (ramping rate reduced to 2%), and three final steps at 4 °C for 5 min, 90 °C for 5 min and a 4 °C infinite hold. Droplet selection was performed individually for each well using quantasoft software v 1.7 (Bio‐Rad, Hercules, CA, USA). Final miRNA amounts (copies·μL^−1^) were obtained and normalized on 50th percentile expression using gx v.14.9.1 software (Agilent Technologies, Santa Clara, CA, USA). None of the candidate reference RNAs included in the plate were used as normalizer due to the higher variability than median expression.

### 2.6 Tissue‐of‐origin prediction

Primary tumors (*N* = 96) were used as training set as previously described [19]. Digital droplet PCR data were normalized on the 50th percentile using genespring gx v.14.9.1 software (Agilent Technologies). Data from primary tumors deriving from the same patient (PF30A/B and PF77A/B) were averaged prior of normalization.

Two different approaches have been applied to select the discriminant miRNAs and to predict the tissue of origin, namely PAM and LASSO. PAM method uses a shrinkage nearest neighborhood centroid approach in the space of the samples. In the training set, PAM calculates centroids as the standardized gene expression within each class (mean divided by standard deviation). Then, a procedure of shrinkage is applied to move centroid toward zero by a quantity called threshold that is set by the user. This threshold is selected based on the results of cross‐validation technique to minimize the error rate. If the shrinkage process reduces to zero the centroid of a gene across all the classes, the gene is not selected for the prediction step. Then, in the prediction step the distance between the expression profile of a new sample with all the class centroid is calculated and the new sample is predicted to belong to the closest one.

LASSO regression is based on a linear regression model where the objective function is penalized by the sum of the absolute value of the parameters. The dependent variable is the class of the samples, and genes are the covariates of the model. The penalization approach has the effect to shrink the parameters estimate toward zero. If the shrinkage procedure set the parameter to zero, the gene will not be used for the prediction. The magnitude of the penalization is selected using cross‐validation technique.

Nearest shrunken centroids (NSC) algorithm [43] using the Prediction Analysis of Microarray for R (PAMR) tool [43] and the least absolute shrinkage and selection operator (LASSO) model [44] were used to build up the classifiers. The PAM threshold was set to 0 leading to a classifier based on 87 miRNAs, while the LASSO threshold was set to 0.019 leading to a classifier based on 53 miRNAs (miRNAs are listed in Table S2). Then, these classifiers were used to predict known and unknown/uncertain metastases tissue of origin. Both predictive models assign to every metastatic tumor a probability to be originated from each primary site. The variable gender was also taken into account to exclude not compatible molecular predictions (TGSC/PRAD in females and OV/UCEC in males). Results were compared with the indications of a possible primary site suggested by standard diagnostic workup and clinicopathological assessment. Bootstrap approach (with *N* = 100) was used to assess the performance (error rate) of the models in the training set.

### 2.7 Cluster analysis

Cluster analysis was performed using the normalized expression (50th percentile) of the 89 miRNAs in (a) individual patients of the reference set of primary tumors and (b) averaged levels within each tumor class of the reference set. The hierarchical cluster analyses were performed using genespring gx v.14.9.1 software (Agilent Technologies) using complete‐linkage rule and Manhattan correlation distance. Standard deviation on the average expression of each miRNA within each class was also assessed.

### 2.8 TCGA data download, filtering, and prediction

Samples from 8 out of 17 tumor types included in this study were present in the TCGA data (BLCA, CHOL, BRCA, LIHC, LUAD, LUSC, OV, PAAD) along with their matched normal tissues. For these eight tumor types and their normal counterpart, we selected our 89 miRNAs using firebrowser R package (MIT, Boston, MA, USA). Of note, we decided to include in this analysis the BRCA class, even though we were aware that it is wider than class. Then, on the whole matrix we applied a two‐step filtering procedure to select samples and miRNAs and eliminate missing values. First, we selected samples with expression values detectable in at least 80% of the miRNA set, and second, we select miRNAs without missing values in the selected sample set. We end up with 835 patients and 48 mRNAs. LIHC tumor samples were excluded from this analysis due to the low quality of these data. The same procedure has been applied for normal tissues obtaining 1533 samples and 47 miRNAs. TCGA data from both normal and tumor samples were used to perform primary site prediction with PAM and LASSO.

### 2.9 Survival analysis

Univariate survival analysis was performed using Kaplan–Meier curves and the log‐rank test, as implemented in survmisc R package. Overall survival (OS) was calculated considering the time lagging between diagnosis and death for any cause or the last follow‐up. For each miRNA, the optimal cut‐off was estimated as the threshold on the ROC curves that maximize the sum of specificity and sensitivity in predicting CUP patients. Results were reported as *P* value, hazard ratio (HR), and 95% confidence intervals (CI). A *P*  value ≤ 0.05 was considered significant.

## 3 Results

### 3.1 Multi‐miRNA testing on archive samples with droplet digital PCR

Formalin‐fixed, paraffin‐embedded tissue is the most commonly available source of tumor material for molecular profiling in the clinical setting, and miRNAs are extremely stable in FFPE blocks. Therefore, we developed an on‐demand multi‐miRNA expression assay capable of testing the absolute levels of 89 miRNAs in a 2‐days timeframe compatible with standard diagnostic workup and with the amount of available material. The multi‐miRNA assay is based on absolute miRNA quantification with EvaGreen Dye Droplet Digital PCR technology [38]. From a technical point of view, the assay provided good quality results for all tested archive FFPE samples. RNA was extracted from 2 to 5 slices of tumor FFPE blocks, and then, the tumor area was identified by experienced pathologists and macrodissected. An amount of 10 ng is sufficient to test all miRNAs in a single experiment, thus confirming the feasibility in a diagnostic setting.

We obtained the absolute copy number for all miRNAs included in our panel in the same droplet digital PCR experiment, with identical experimental conditions (annealing temperature and amount of primers), only adjusting the amount of input cDNA for miR‐21‐5p and UniSP6.

With the aim of establishing a reference set for cancer of unknown origin molecular profiling, we tested 96 primary tumors with our multi‐miRNA assay, comprising 16 different tumor types and 19 histological classes, focusing on the most common CUP's sites of origin identified at autopsy [45]. We obtained the expression matrix of the primary tumor dataset, constituted by tumors belonging to 19 different classes: LUAD, LUSC, PAAD, LIHC, CHOL, KIRC, KIRP, STAD, CRC, TGSC, OV, UCEC, BLCA, LBC, TNBC, PRAD, SKCM, GI‐NET, and HNSC. An overview of the primary tumor samples for each histological subtype included in this study is reported in Table 1.

### 3.2 Analysis of miRNA expression patterns

**Fig. 1:**
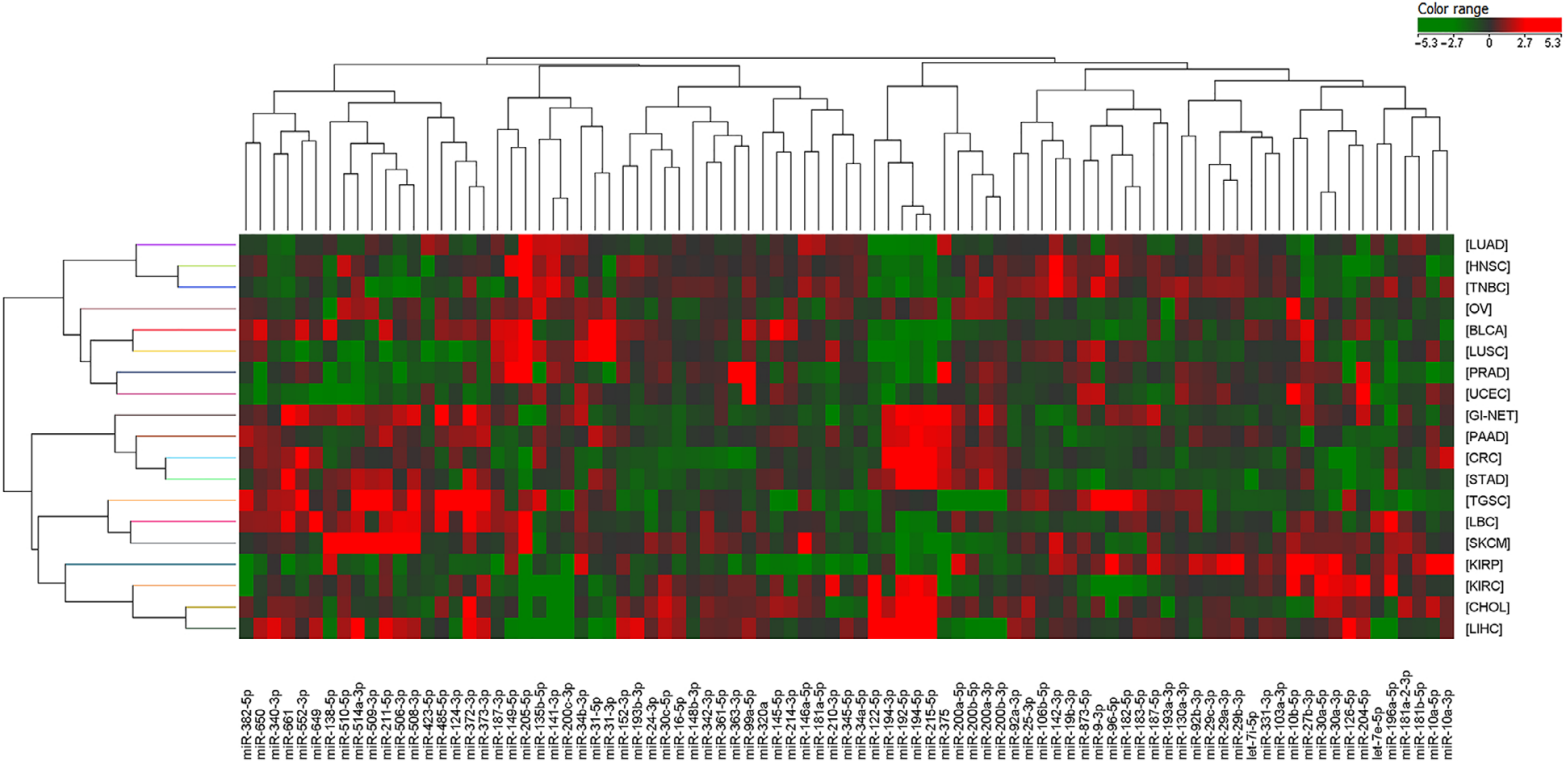
Cluster analysis of primary tumors. Heatmap representing the expression of 89 microRNAs in 19 different classes of primary tumors. Averaged, normalized miRNA levels in each tumor class were used for clustering analysis. Green indicates low expression, and red indicates high expression.

We evaluated the average levels of normalized expression of the 89‐miRNA signature in the nineteen primary tumor types with cluster analysis (Fig. 1). Average miRNA expression and standard deviations within each cancer type are reported in Table S3. Clustering analysis of individual patients belonging to the reference set (*N* = 94) is reported in Fig. S1. Each tumor type displays a peculiar pattern of miRNA expression, as expected. Nonetheless, we found some unexpected similarities and divergences among tumor types, which are worth mentioning. Specifically, miRNA expression of STAD and CRC was found to be consistently overlapping and partially intermixed with other gastrointestinal tumors (PAAD and GI‐NET), as reported also in previous reports [19, 39, 46]. Due to this miRNA expression similarity, we decided to consider them as a single class (STAD‐CRC) for molecular prediction. Similarly, kidney renal clear cell (KIRC) and papillary cell carcinomas (KIRP), showing similar miRNA expression patterns, were combined in the tumor class KICA. Tumors in female reproductive‐system organs (OV and UCEC) were found to express similar yet distinct miRNA patterns as previously observed [19, 39, 46]. Moreover, lung cancers (both LUAD and LUSC) share a portion of their signatures with TNBC but not with other breast cancer subtypes (ER+, PR+, HER2+ tumors). TNBC shows a largely different pattern of miRNA expression when compared to other breast cancers, showing an unexpected similarity with HNSC instead. We could speculate that a common etiology associated to human papillomavirus (HPV) infection has been reported in both these tumor types [47, 48, 49]. Overall, this signature confirmed its potential in discriminating among 17 different tumor classes.

### 3.3 CUP predictive model generation

The final primary site prediction was performed using 87 out of 89 miRNA assays of our panel. Among the two miRNAs excluded from the prediction analysis, miR‐122‐5p was omitted due to its strong signal generated by the liver microenvironment in metastatic samples (Fig. S2), while miR‐21‐5p was excluded from the analysis due to its lack of specificity with both classifiers (it is widely expressed in solid tumors).

We applied the nearest shrunken centroids (NSC) using PAMR [43] and the least absolute shrinkage and selection operator (LASSO) predictive models [44] developed by Tibshirani's laboratory to our training set of primaries. To assess the performance of the predictive models on the training set, we used a bootstrap approach. Error rates for each tumor class for both models are reported in Table S4. Notably, the overall error rate for both PAMR and LASSO was 33%. However, 11 of the 17 tumor classes (LIHC, LUSC, LBC, KICA, GI‐NET, TGSC, STAD‐CRC, SKCM, LUAD, UCEC, and PRAD) had error rates much lower with both models (17% for PAMR and 22% for LASSO). Of note, PAMR seems to be considerably more accurate in the prediction of LBC and LUSC compared to LASSO; on the contrary, LASSO seems to be more precise in the identification of UCEC, LUAD, and SKCM. Both models had higher error rates in identifying correctly BLCA, PAAD, TNBC, HNSC, OV, and CHOL classes; this might be explained by the reduced specificity of the miRNA signature for these primaries and cross‐prediction (e.g., CHOL and PAAD or TNBC and HNSC) or the smaller sample size of TNBC (*n* = 3) and BLCA (*n* = 4). From these results, it is clear that the two models behave similarly on some classes and complementarily in some others; therefore, we decided to take advantage of both classifiers and combine their molecular prediction.

A small set of metastases of known origin (*N* = 10) was assessed for molecular prediction (test set Table 1). Considering the two top predicted classes, we obtained an accuracy of 80% for PAMR and of 60% for LASSO, as reported in Table S5.

In addition, we evaluated the ability of our signature to correctly classify primary tumors belonging to eight classes included both in our study and TCGA database, specifically BLCA, BRCA, CHOL, LIHC, LUAD, LUSC, OV, and PAAD. In this validation, we included both tumor and matched normal samples. miRNA expression data in TCGA classes were available for 48 miRNAs in tumor samples and 47 miRNAs in normal samples with adequate quality signal. PAMR and LASSO predictions showed an overall median positive prediction rate (PPR) higher than 80% for both tumor and normal samples (Table S6).

### 3.4 CUP primary site prediction

**Fig. 2:**
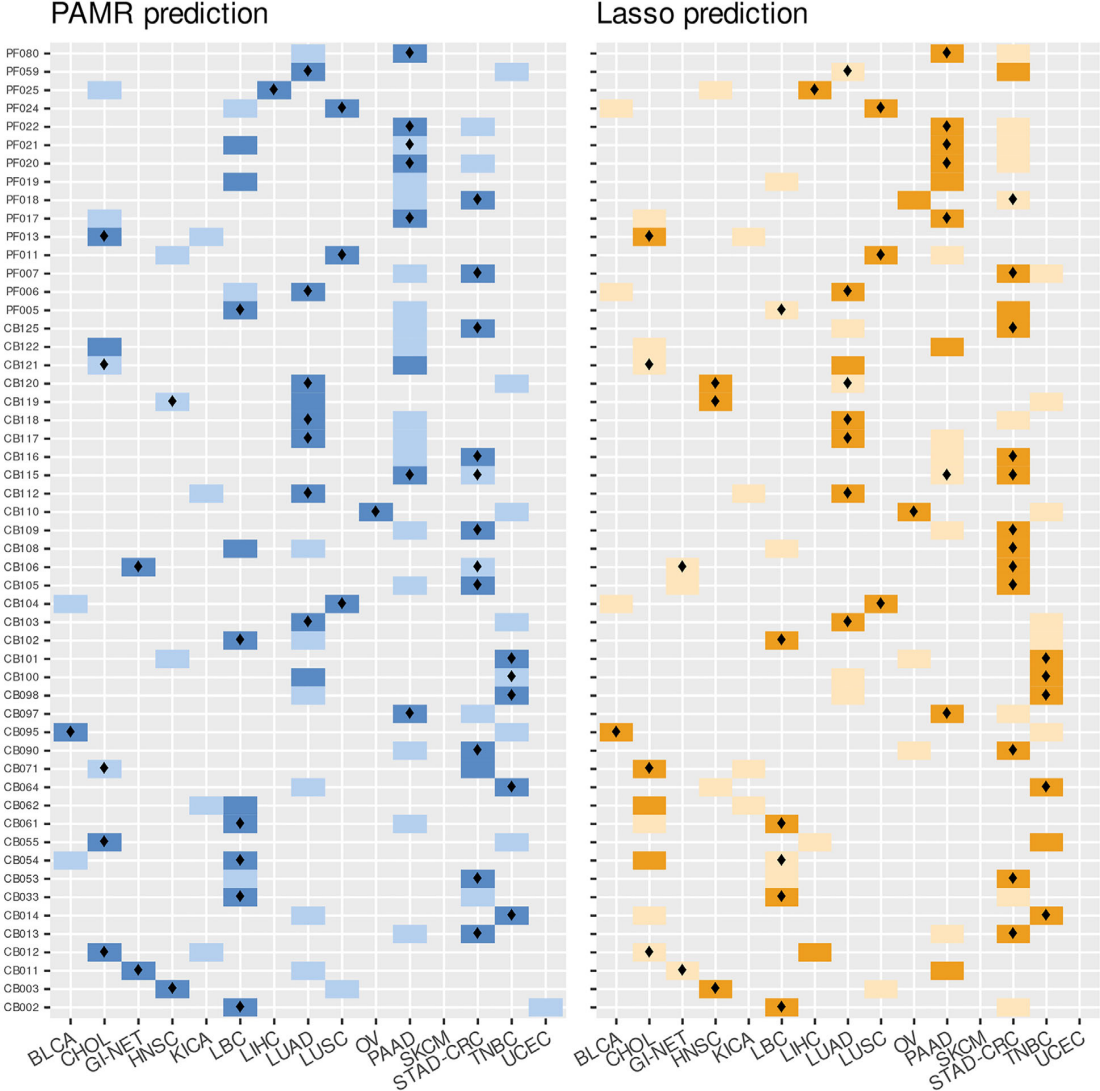
Prediction outcome of cancers of unknown origin using PAMR NSC and LASSO classifiers. For each of the 53 CUP sample (on the *y*‐axis), the two top predicted primary tumors (*x*‐axis) are highlighted. PAMR first and second molecular predictions are reported with dark and light‐blue squares, respectively. LASSO first and second molecular predictions are reported in dark and light orange, respectively. A diamond in the cell indicates those tissues of origin that are consistent with pathological and/or clinical information. BLCA, transitional cell carcinoma of bladder; CHOL, cholangiocarcinoma; STAD‐CRC, colorectal and gastric adenocarcinoma; GI‐NET, gastrointestinal neuroendocrine carcinoma; HNSC, head and neck squamous cell carcinoma; KICA, kidney renal clear and renal papillary cell carcinoma; LBC, luminal nonspecial type and lobular breast carcinoma; LIHC, hepatocellular carcinoma; LUAD, lung adenocarcinoma; LUSC, lung squamous cell carcinoma; OV, ovarian serous carcinoma; PAAD, pancreas exocrine adenocarcinoma; SKCM, melanoma of skin; TNBC, triple‐negative breast cancer; UCEC, endometrial adenocarcinoma.

Finally, both models were used to predict the primary site of 53 cancers of unknown/uncertain origin (CUPs). Given the tumor frequency, this is a remarkably large collection of cancers of unknown primary site whose histopathological and immunohistochemistry characteristics are detailed in Tables 2 and S1. The prediction outcome is represented in Fig. 2 in which the top two primary sites predicted by both models for each CUP sample are reported. Using PAMR, the molecular prediction of 43 out of 53 CUPs (81%) reached a probability higher than 60%, 55.8% of them even higher than 90%. Using LASSO, the molecular prediction of 25 out of 53 CUPs (47.2%) predicted the first primary site with a probability higher than 60% and 7 higher than 90%.

The most probable primary sites, reported in Table 2, were prioritized using the following criteria: (a) The primary site was predicted by at least one predictive model (LASSO or PAMR) with a probability higher than 80%, and (b) the primary site was present among the predicted sites in both models, with a probability higher than 30% in at least one prediction. If the prediction outcome did not fall within these criteria, we reported all the predicted primary sites (including the first and second predictions). Following the prioritization, a probable tissue of origin was assigned to each CUP. Few cases had more than one tissue of origin. Of note, a high agreement was observed between PAMR and LASSO predictions: Specifically, the same primary sites (according to the above‐mentioned criteria) were predicted by both models in 94% of cases.

We also evaluated the compatibility of this molecular prediction with the clinicopathological information available. Final predictions were found in agreement with the first hypothesis of a primary site in 53% of CUPs in which a hypothesis was made. In addition, in those patients in which the primary site was later identified (*N* = 3, CB071, CB054, and CB098/100/101/102), we observed a 100% concordance between the diagnosis and the molecular prediction.

We identified a subgroup of CUP samples (*N* = 5) for which it was very challenging to point out a tissue of origin using both models, with molecular predictions with a probability ≤ 40%. These could derive from patients characterized by an exceptionally undifferentiated phenotype or could also derive from tissues of origin not included in the reference set. Considering the final predicted sites reported in Table 2, the most common tissues of origin were STAD‐CRC (19%), LBC (15%), PAAD (15%), LUAD (13%), CHOL (11%), LUSC (5%), TNBC (8%), and HNSC (5%) and others at lower rates. Of note, no CUP was predicted to originate from TGSC or PRAD.

Interestingly, from five CUP patients we obtained a number of samples (*N* = 2–4) derived from spatially distinct synchronous and metachronous metastases, which were all tested with our assay. These samples were used to evaluate the consistency of our prediction and its independence from the metastatic site. Symbolic is the case of a patient (#B) with an initial diagnosis of CUP (later attributed to a breast origin) from which we obtained a total number of four samples (CB098, CB100, CB101, and CB102). In particular, CB098 and CB100 were obtained from two lymph nodes resected in 2010, while CB101 and CB102 derived from an invasive ductal breast cancer identified two years later, which was recognized as the primary site. Both PAMR and LASSO agreed to predict it as a LBC or TNBC (Table 2). However, CB100 was predicted as LUAD (first) or TNBC (second) by PAMR classifier (Fig. 2), probably due to the lower tumor cell fraction in this sample and the reported similarity in miRNA expression between breast and lung cancers [19]. Molecular predictions for the multiple metastases of the other patients (#E, #F, #Q, and #R) reported concordant results for both models, in agreement with clinicopathological hypotheses. Moreover, for #E (CB105 and CB106) and #F (CB108 and CB109) both models agreed to predict a gastrointestinal origin (STAD‐CRC), which was also the first clinicopathological hypothesis. CB108 from #F patient had a different indication as the most probable tissue of origin with PAMR classifier (LBC); however, being derived from the bone it is probable that the sample had a compromised integrity. Molecular prediction for #R (CB121 and CB122) pointed out to a biliopancreatic origin, while for #Q (CB119 and CB120), the two metastatic samples were predicted to have the same origin, which was in this case lung or head and neck. CUP prediction probabilities with PAMR and LASSO models are reported in Table S7.

### 3.5 Association of microRNAs with CUP patients' overall survival

**Fig. 3:**
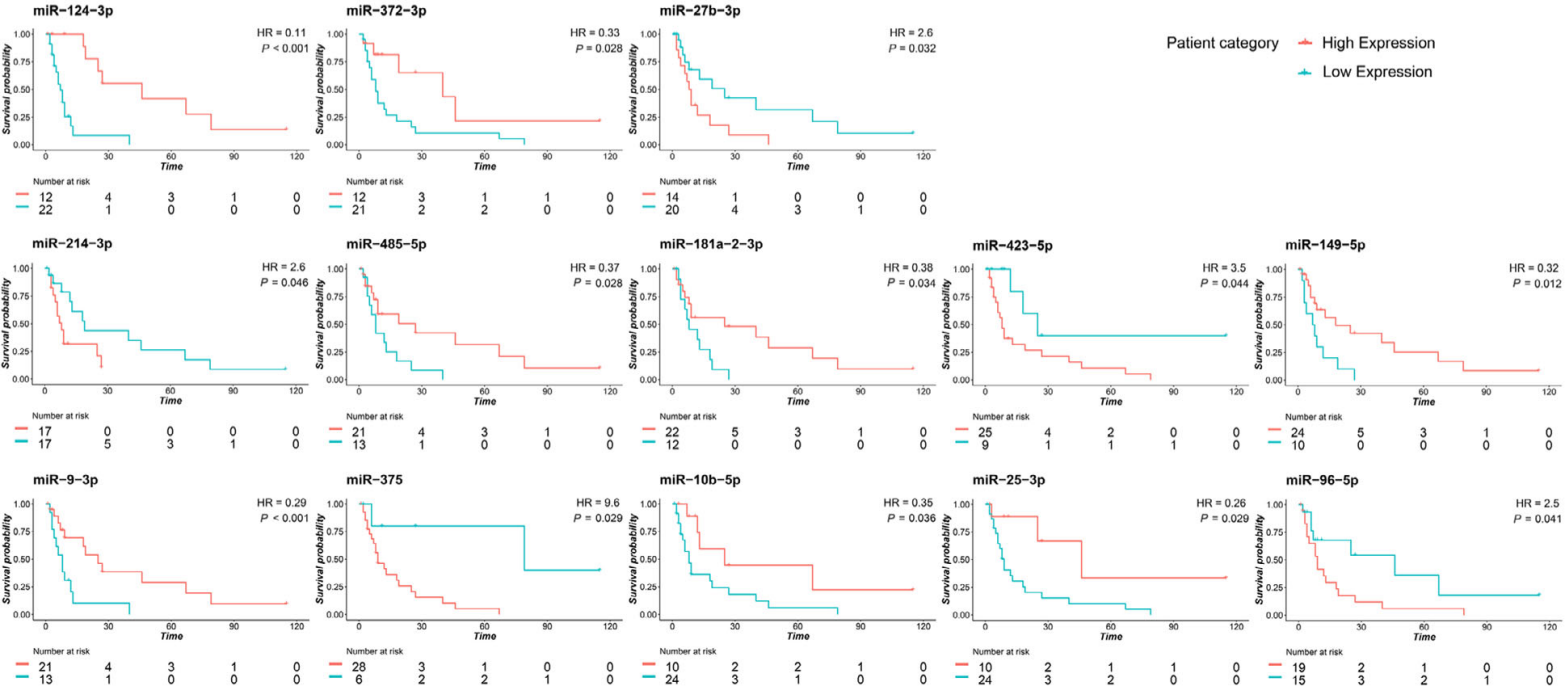
Kaplan–Meier OS curves based on the expression of 13 miRNAs in CUP patients. Survival plots showing significantly different OS curves in high and low miRNA expressing CUPs. The log‐rank test was used to compare the survival distributions. The threshold for each miRNA was established based on the best performing value at ROC analysis. For five miRNAs, a higher expression is associated with shorter CUP survival, and for eight miRNAs, a higher expression is associated with prolonged survival. The *x*‐axis represents the months from the diagnosis.

We tested the performance of our 87‐miRNA panel as prognostic test for CUP patients. Survival information was available for 34 CUP patients included in this study. We performed a survival analysis to test the association of miRNA expression with overall survival (Table S8) finding 13 miRNAs with significant prognostic effect on CUP patients' OS (Table 3 and Fig. 3). The association between survival probability and miRNA expression was negative for five miRNAs (HR > 1) and positive for eight miRNAs (HR < 1).

**Table 3:** Association of miRNA expression with overall survival (significant miRNAs). For each miRNA, the hazard ratio (HR) with 95% confidence interval and *P*‐value is reported for OS.

miRNA	HR	Lower 95%	Upper 95%	*P*‐value
miR‐124‐3p	0.11	0.03	0.36	0.00
miR‐9‐3p	0.29	0.12	0.71	0.01
miR‐149‐5p	0.32	0.13	0.78	0.01
miR‐372‐3p	0.33	0.12	0.89	0.03
miR‐485‐5p	0.37	0.16	0.90	0.03
miR‐375	9.60	1.30	73.00	0.03
miR‐25‐3p	0.26	0.08	0.87	0.03
miR‐27b‐3p	2.60	1.10	6.10	0.03
miR‐181a‐2‐3p	0.38	0.15	0.93	0.03
miR‐10b‐5p	0.35	0.13	0.93	0.04
miR‐96‐5p	2.50	1.00	6.20	0.04
miR‐423‐5p	3.50	1.00	12.00	0.04
miR‐214‐3p	2.60	1.00	6.60	0.05

In particular, the miRNAs whose higher expression is associated with worse prognosis are miR‐375 (*P* = 0.03), miR‐27b‐3p (*P* = 0.03), miR‐96‐5p (*P* = 0.04), miR‐423‐5p (*P* = 0.04), and miR‐214‐3p (*P* = 0.05). On the contrary, eight miRNAs are positively associated with a prolonged survival: miR‐124‐3p (*P* = 0.0002), miR‐9‐3p (*P* = 0.01), miR‐149‐5p (*P* = 0.01), miR‐372‐3p (*P* = 0.03), miR‐485‐5p (*P* = 0.03), miR‐25‐3p (*P* = 0.03), miR‐181a‐2‐3p (*P* = 0.03), and miR‐10b‐5p (*P* = 0.04).

## 4 Discussion

The identification of the tissue of origin in metastatic cancers strongly relies on clinical information and histology as well as immunohistochemical evaluations but this diagnostic workup is sometimes ineffective and a fraction of primaries remains unidentified. Epitome of this scenario is metastatic cancer of unknown primary site (CUP), which presents by definition as an advanced cancer whose site of origin is not detectable nor presumable, despite an intensive clinical and pathological diagnostic workup [1]. CUPs represent an enigma at both biological and pathological levels and an important under‐researched clinical problem.

In the past decade, several molecular tests based on gene expression (GEP), microRNA, or DNA methylation profile were developed to improve primary site identification in cancers of unknown/uncertain origin. The underlying premise for these molecular profiling assays (reviewed in Refs [50] and [51]) is that metastatic tumors preserve specific molecular signatures that match their primary site and can be used to identify their site of origin.

Overall, these methods reach a prediction accuracy that ranges from 80% to 95% and have the potential to improve the diagnostic workup of CUP patients and guarantee the access to more therapeutic options. Indeed, NCCN occult primary guidelines recently assessed CUP molecular profiling as a potential provider of clinical benefit for patients. At the present time, CUP molecular profiling's clinical utility needs to be determined on a case‐by‐case basis, and clinical validation in large randomized phase III trials is still missing.

In this study, we developed a molecular assay to assess the expression of 89 miRNAs in tumor FFPE samples by using droplet digital PCR (ddPCR) and infer CUP primary site [38]. Our miRNA panel was determined merging two cancer‐specific miRNA signatures previously identified in two microarray‐based studies [19, 39]. To prevent the costs of large‐scale technologies such as microarrays or sequencing, we opted for a focused number of selected miRNAs and the use of ddPCR technology. This assay allows the on‐demand quantification of a focused panel of miRNAs per sample, at an affordable cost and in a 2‐day timeframe. Droplet digital PCR technology provides miRNA absolute quantification without the requirement of standard curves, efficiency correction approaches, or technical replicates typical of traditional quantitative PCR approaches [52]. In addition, EvaGreen‐based ddPCR allows to precisely detect target miRNAs at levels down to 1 copy·μL^−1^ [53].

As we hypothesized, an approach based on miRNA expression instead of gene expression profiles is very convenient since we were able to successfully analyze the totality of FFPE samples in our cohort (100% success rate), with no excluded sample due to technical issues.

In this study, we analyzed the 89‐miRNA profiles of 159 FFPE samples, including 53 CUPs, and successfully obtained a primary site prediction for all patients. We obtained a good prediction accuracy rate in metastatic cancers of known origin and highly consistent results when assessing multiple metastases derived from the same CUP patient. These two settings provided an intrinsic validation of our combined predictive models.

As for CUP predictions, we observed consistency between our prediction outcomes and clinical and histopathological hypotheses, when they were available. In addition, we were able to successful analyze all 159 FFPE samples, with no excluded sample due to technical issues. The employment of two predictive models allowed us to obtain stronger results when both systems pointed out to the same tissue of origin. Of note, some CUPs were molecularly predicted as LUAD with a negativity for TTF1, which defines a subgroup of LUAD with unfavorable outcomes [54]. Our results provide further evidence of the translational potential of CUP molecular testing in general and miRNA testing in particular. With no intention to replace IHC testing, molecular assays can support the pathologists in narrowing the spectrum of possible primary sites of undifferentiated metastatic tumors. When no pathological hypothesis can be formulated, the miRNA‐based molecular assay could aid the oncologists in their therapeutic choice, despite being necessary to demonstrate a benefit in a clinical setting.

The droplet digital PCR, miRNA‐based assay herein applied has an accuracy comparable with other commercialized molecular profiling assays, but overcomes some limits of previous tools. Our molecular classifiers have the advantage to cover a wide variety of primary cancers, among the most likely to be CUP's sites of origin; in particular, we can discriminate between 17 primary tumor subtypes. The ability to cover such number of tumor classes is an advantage if compared to other commercialized molecular assays, for example, the 10‐gene qPCR assay (Veridex, Raritan, NJ, USA), that can classify only six different tumor types. Our prediction outcome on CUPs mostly overlaps the frequency rates identified in postmortem autopsy studies: lung (27%), pancreas (24%), liver or bile duct system (8%), kidney or adrenal (8%), or colon (7%) [45].

Three molecular assays were recently approved for CUP diagnostics in US: Pathwork Tissue of Origin Test (Pathwork Diagnostics, Redwood City, CA, USA), CancerTYPE ID (bioTheranostics, San Diego, CA, USA), and miRview mets^2^ (Rosetta Genomics, North Brunswick, NJ, USA). The first is a microarray‐based system to assess the gene expression profiles (GEP) of 2000 genes claim to distinguish up to 15 tumor types. CancerTYPE ID is another GEP‐based assay which evaluates by RT‐qPCR the expression of a 92‐gene signature and identifies the primary origin of up to 30 tumor types. Finally, miRview mets^2^ system, assessing the expression of 64 miRNAs by RT‐qPCR, is able to distinguish up to 26 tumor types.

However, these assays included primary tumors that have little or no connection with CUPs. Our molecular tool is able to cover a high number of tumor classes, selected as the most common CUP tissues of origin. Our assay has a 100% success rate and requires a 2‐day working time, which is compatible with a standard diagnostic workup and consistently shorter compared to other commercial assays that present a turnaround time of 5–11 days.

In addition to being faster, targeted, and cost‐effective in primary site identification, our assay could be easily combined with the analysis of druggable alterations, to select CUP therapy. However, further prospective clinical studies are necessary to evaluate their use in the clinics and to demonstrate its possible impact on CUP patients' survival.

## 5 Conclusions

In conclusion, our study demonstrated that digital miRNA expression profiling of CUP samples has the potential to be employed in a clinical setting in FFPE tissue. Our molecular analysis can be performed on request, concomitantly with the standard diagnostic workup and in association with genetic profiling, to offer valuable indication about the possible primary site thereby supporting treatment decisions.

## Conflict of interest

AA received honoraria (self) for advisory board participation: BMS, MSD, ROCHE, AstraZeneca, Eli‐Lilly‐Research Grants to my Institution: Celgene, BMS, Ipsen, Roche. The remaining authors have no conflicts of interest to declare.

## Author contributions

MF conceived and designed the study. NL, MR, EP, MF, and CR developed the methodology. IG, FV, AAi, ND, IBN, AAr, DT, and AD acquired the data. NL, MR, EP, FA, GD, GB, SS, AD, MP, MF, and CR analyzed and interpreted the data. NL, MR, MP, AD, and MF wrote, reviewed, and/or revised the manuscript. MF supervised the study.

## Consent for publication

All authors of the manuscript have read and agreed to its content.

### Peer Review

The peer review history for this article is available at https://publons.com/publon/10.1002/1878‐0261.13026.

## Supporting information

**Fig. S1.** Clustering analysis on individual patients of the training set. Heatmap representing the expression of 89 microRNAs in 94 samples of the training set belonging to nineteen different classes of primary tumors. Normalized miRNA levels for each sample were used for clustering analysis. Green indicates low expression, red indicates high expression.

**Fig. S2.** Plot of miR‐122‐5p expression in primary and metastatic tumors. Normalized miR‐122‐5p expression was evaluated in liver and bile duct primary tumors, known to express this miRNA at high levels, and in metastatic tumors of known/unknown origin whose biopsy was obtained from the liver tissue or other sites. Liver metastases of known/unknown origin show high levels of miR‐122‐5p if compared to those derived from other sites, which is due to the very abundant expression of miR‐122 in liver cells and its release in the tumor microenvironment.

**Table S1.** Clinic‐pathological features of 159 samples.

**Table S2.** List of miRNA assays in the custom ddPCR plate.

**Table S3.** Average miRNA expression and standard deviations for each tumor class.

**Table S4.** Error rates of the PAMR and LASSO models for each tumor class.

**Table S5.** Primary site prediction in metastases of known origin.

**Table S6.** Confusion matrix of LASSO and PAMR in tumor and normal tissue based on TCGA data.

**Table S7.** CUP probabilities with PAMR and LASSO classifier models.

**Table S8.** Association of miRNA expression with CUP overall survival (all miRNAs).

## Data Availability

Droplet digital PCR data are available upon request.
