# Supplementary figures and images for: MicroRNA expression profiling with a droplet digital PCR assay enables molecular diagnosis and prognosis of cancers of unknown primary
Source: Mol Oncol. 2021 Jun 23;15(10):2732–51. doi: 10.1002/1878-0261.13026 (PMC8486570; doi:10.1002/1878-0261.13026)

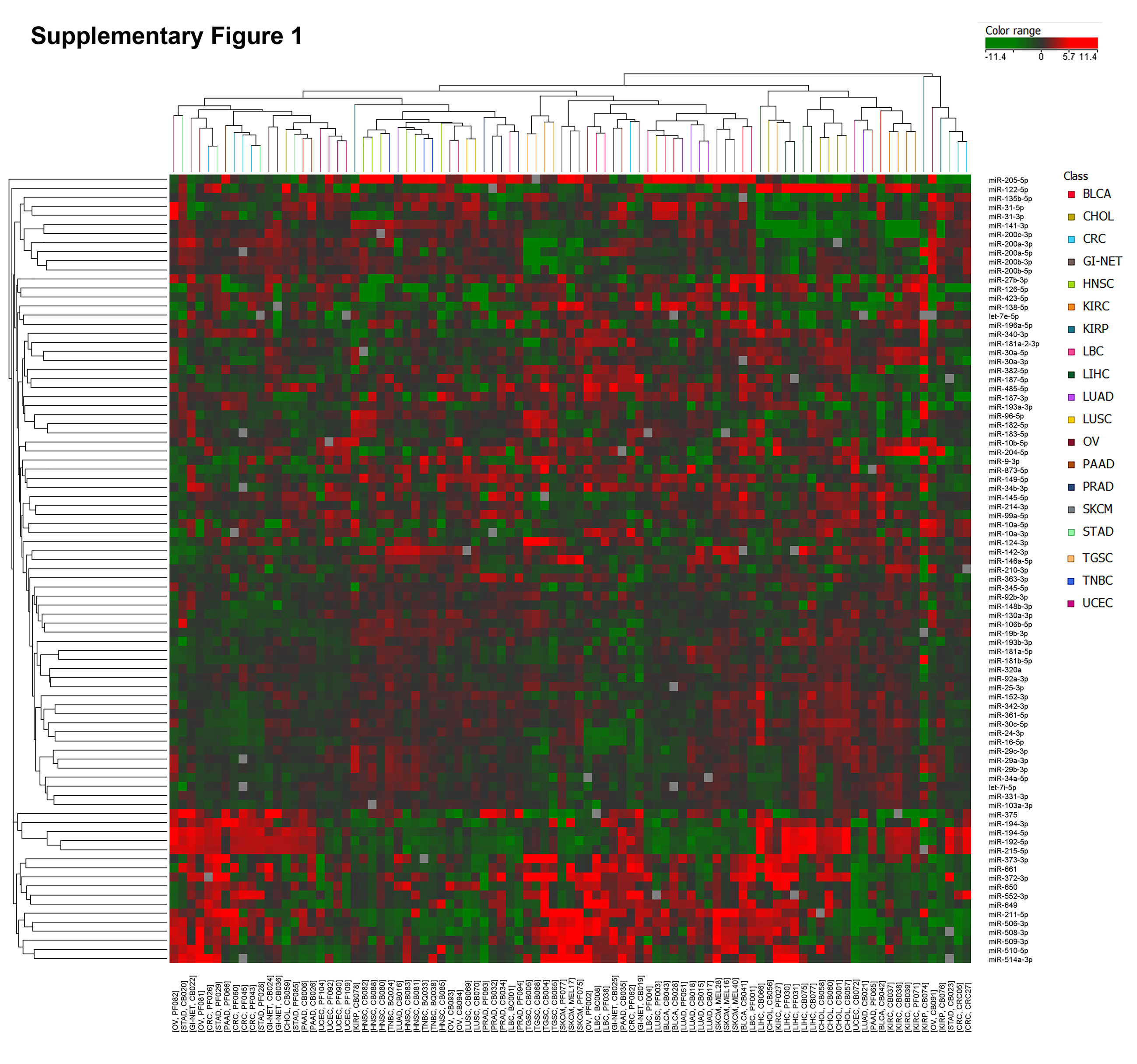

Supplement: Supplementary file 1 — Fig. S1. Clustering analysis on individual patients of the training set. Heatmap representing the expression of 89 microRNAs in 94 samples of the training set belonging to nineteen different classes of primary tumors. Normalized miRNA levels for each sample were used for clustering analysis. Green indicates low expression, red indicates high expression. [file MOL2-15-2732-s004.tif]

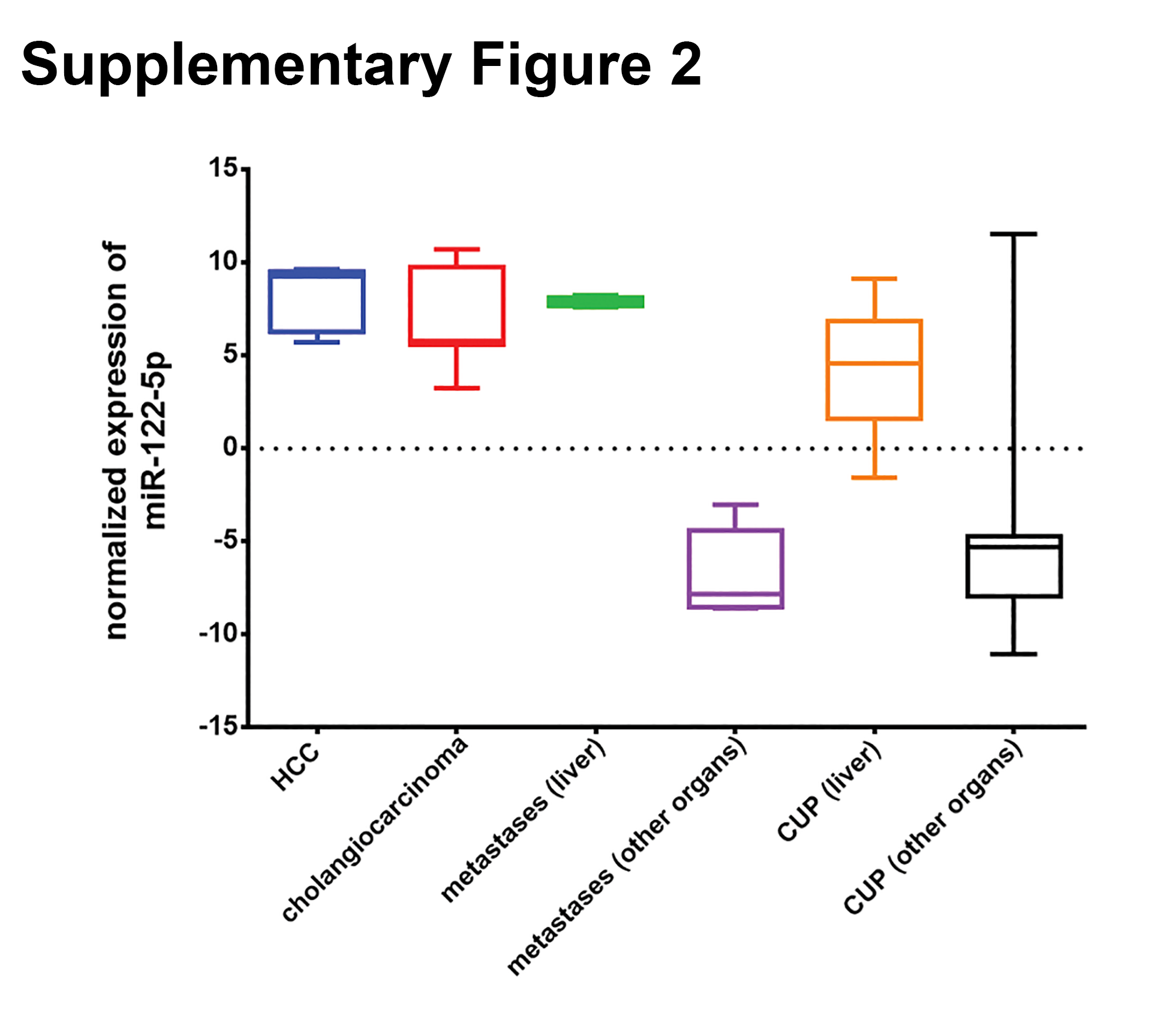

Supplement: Supplementary file 2 — Fig. S2. Plot of miR‐122‐5p expression in primary and metastatic tumors. Normalized miR‐122‐5p expression was evaluated in liver and bile duct primary tumors, known to express this miRNA at high levels, and in metastatic tumors of known/unknown origin whose biopsy was obtained from the liver tissue or other sites. Liver metastases of known/unknown origin show high levels of miR‐122‐5p if compared to those derived from other sites, which is due to the very abundant expression of miR‐122 in liver cells and its release in the tumor microenvironment. [file MOL2-15-2732-s001.tif]
